# Supplement Safety Knowledge, Information Sources, and Self-Reported Safety-Related Behaviors Among Adults Aged 40 Years and Older: A Pilot Study

**DOI:** 10.3390/nu18172921

**Published:** 2026-09-07

**Authors:** Beata Walczyk, Agnieszka Białek

**Affiliations:** 1 School of Health and Medical Sciences, VIZJA University, Okopowa 59, 01-043 Warsaw, Poland; 2 The Kielanowski Institute of Animal Physiology and Nutrition, Polish Academy of Sciences, Instytucka 3, 05-110 Jabłonna, Poland

**Keywords:** dietary supplements, supplement safety, supplement–medicine interactions, health knowledge, information sources, healthcare professionals, poly-supplementation, pilot study

## Abstract

Background/Objectives: Dietary supplement use is common in middle and later adulthood, but factual safety knowledge may not necessarily translate into safety-related behavior. This pilot study examined supplement use, supplement safety knowledge, information-source pathways, and self-reported safety-related behaviors among adults aged 40 years and older. Methods: A cross-sectional questionnaire study was conducted in Poland between December 2025 and April 2026 in a convenience sample of 150 adults aged ≥40 years. Multivariable logistic regression models assessed associations of a five-item factual supplement safety knowledge score and professional or digital information-source use with proactive supplement safety information seeking and other supplementation behaviors. Results: Current supplement use was reported by 113/150 participants (75.3%); among current users with available supplement count data, 86/111 (77.5%) used at least two supplements simultaneously. The knowledge score was not independently associated with proactive supplement safety information seeking (aOR 0.93 per one-point increase, 95% CI 0.69–1.25; *p* = 0.641). Professional information-source use was associated with the original composite information-seeking outcome (aOR 4.03, 95% CI 1.51–10.77; BH q = 0.033) and with lower odds of self-initiated supplementation (aOR 0.32, 95% CI 0.13–0.79; BH q = 0.042). However, in an additional non-overlap sensitivity analysis restricted to internet/leaflet safety information seeking, professional-source use was not independently associated with the outcome (aOR 0.80, 95% CI 0.33–1.94; *p* = 0.627). Conclusions: In this convenience sample, the study-specific factual knowledge score did not show a clear independent association with proactive supplement safety information seeking. Associations involving professional information sources depended partly on outcome definition, while the association with lower self-initiation remained. Given the pilot nature, modest sample size, non-probability recruitment, self-reported measures, and lack of product-level verification, the findings are hypothesis-generating and require confirmation in larger, prospectively characterized samples.

## 1. Introduction

Dietary supplement use is reported across adult populations, although prevalence estimates vary with age, population characteristics, definitions of use, and methods of assessment. Polish population-based studies have documented substantial supplement use together with heterogeneity in consumption patterns and consumer awareness [1,2]. Use is also reported in middle-aged and older populations: more than four in five community-dwelling U.S. adults aged ≥50 years in a nationally representative sample reported using at least one dietary supplement [3], whereas a nationally representative random sample of 5987 Polish adults aged ≥60 years reported a prevalence of 32.1%, with use varying according to sociodemographic and health-related characteristics [4]. These differences underscore the importance of interpreting supplement use estimates within the characteristics and sampling framework of each study.

Supplement safety is particularly relevant when multiple products are used concurrently or when supplementation coexists with pharmacotherapy. A systematic review in older adults documented concurrent use of prescription medicines and herbal medicinal products and identified potentially relevant interactions [5]. A broader systematic review identified 1491 documented herb– or dietary supplement–drug interaction pairs involving 213 supplements or herbal entities and 509 medications [6]. More recent evidence has continued to highlight the relevance of supplement use in older adults, in whom supplementation may coexist with polypharmacy and increase the potential for drug–supplement and supplement–supplement interactions, as well as duplicate exposure [7]. In addition, analysis of nationally representative U.S. surveillance data estimated approximately 23,000 emergency department visits annually for adverse events associated with dietary supplements [8]. These findings provide a rationale for examining self-reported behaviors related to recommended dosing, professional oversight, and checking for potential supplement–medicine interactions or adverse effects, particularly in adults for whom medication use may coexist with supplementation.

Knowledge is one potential component of supplement-related decision-making, but available evidence indicates that knowledge, attitudes, intentions, and behavior are not interchangeable constructs. In a nationwide study of Polish Internet users, dietary supplement knowledge was associated with individual characteristics, beliefs, attitudes, and information-related factors [9]. Using the theory of reasoned action, Bayır et al. examined food supplement safety knowledge within a broader framework that included health consciousness, safety concerns, attitudes, subjective norms, and behavioral intentions [10]. In a nationwide Polish survey focused on supplements for eye health, supplement use was not fully aligned with respondents’ self-reported knowledge, and medical recommendation was only one of several motivations for use [11]. Collectively, these studies support examining factual knowledge and reported supplementation behaviors as related but distinct domains.

Information sources represent a further component of supplement-related decision-making. Consumers may obtain supplement information from physicians, pharmacists, dietitians, family or peers, traditional media, websites, social media, and online discussion groups. In Poland, the Internet has been reported as a major source of dietary supplement information, while healthcare professionals also remained important sources, and patterns of source use differed across sociodemographic groups [2]. International evidence similarly indicates reliance on multiple professional and non-professional information channels [12]. Importantly, frequency of source use does not necessarily correspond to perceived trustworthiness: in an international survey, online sources were frequently consulted for diet and nutrition information, whereas nutrition professionals, scientists, and scientific literature were among the most trusted sources [13]. Recent research among dietary supplement users has likewise emphasized the importance of information behavior alongside motivations, perceptions, and intentions [14]. Professional and digital sources may therefore represent distinct information pathways, but cross-sectional source-use data cannot establish whether use of a given source precedes, follows, or contributes to a particular supplementation behavior.

Previous studies have commonly examined supplement prevalence, knowledge, attitudes, motivations, behaviors, or information sources as separate domains [1,2,9,10,11,12,13,14]. Fewer studies have integrated factual supplement safety knowledge, professional and digital information-source use, and specific self-reported safety-related supplementation behaviors within the same analytical framework. An important question is whether a measured factual knowledge score is independently associated with proactive checking for potential supplement–medicine interactions or adverse effects, and whether professional and digital source use show distinct cross-sectional associations with such checking, professional oversight, self-initiation, and poly-supplementation. The present pilot study addresses this gap by evaluating these domains together in a convenience sample of Polish adults aged ≥40 years.

Accordingly, the primary aim was to characterize dietary supplement use, factual supplement safety knowledge, information-source pathways, and self-reported safety-related supplementation behaviors and to examine their cross-sectional associations. The primary hypothesis was that a higher five-item factual supplement safety knowledge score would be associated with greater odds of proactive checking for potential supplement–medicine interactions or adverse effects. We further hypothesized that professional information-source use would be positively associated with proactive checking and professional oversight and negatively associated with self-initiated supplementation after adjustment for prespecified demographic and health-related covariates. Associations involving digital information-source use, poly-supplementation, participation in the Polish ‘Profilaktyka 40+’ preventive health program, and empirically derived behavioral profiles were treated as secondary or exploratory analyses.

## 2. Materials and Methods

### 2.1. Study Design and Ethical Considerations

This pilot cross-sectional questionnaire study was conducted among adults aged ≥40 years residing in Poland. Data were collected between December 2025 and April 2026. The study was voluntary and anonymous, and participants were informed about the purpose of the study, the scientific use of the data, and their right to discontinue participation. The questionnaire did not request direct personal identifiers. The study was conducted in accordance with the Declaration of Helsinki and was approved by the Research Committee of VIZJA University (Resolution No. W027.01122025.P, 22 December 2025). Informed consent was obtained before participation.

### 2.2. Participants and Recruitment

Eligible participants were adults aged ≥40 years who resided in Poland and provided informed consent. Recruitment used convenience sampling. The questionnaire was administered in both electronic and paper formats. The electronic questionnaire was created using Microsoft Forms, and the survey link was disseminated through social media, including Facebook and Instagram. Paper questionnaires were distributed directly to individuals meeting the inclusion criteria. Responses collected using the paper version were manually entered into the study Microsoft Excel database by B.W., and the accuracy of transcription was independently checked by A.B. against the original paper questionnaires. The electronic and paper versions used the same questionnaire content and response structure. The source workbook contained 156 response records. Application of the reproducible eligibility rules yielded a final analytical cohort of 150 participants. Six source records were excluded because the age eligibility criterion was not met. The planned minimum sample size in the original study documentation was 100 respondents; no formal a priori power calculation was performed. Given the convenience-sampling design and modest sample size, the present work is framed as a pilot study rather than as a population-representative survey.

### 2.3. Questionnaire Development and Administration

The author-developed questionnaire was prepared specifically for the study following a review of the literature, alignment with the study objectives, and consultation with the experts. Selected elements of structure and question format were informed by the KomPAN questionnaire, whereas questions concerning dietary supplements, supplement safety, and the ‘Profilaktyka 40+’ program were study-specific. The questionnaire contained 46 numbered questions organized into eight thematic sections and included single-choice, multiple-choice, semi-open and open questions, five-point Likert-type items, and factual true/false/don’t-know statements. The questionnaire was designed to require approximately 10–15 min to complete. Before the main survey, the instrument underwent content review by the experts and pilot testing with 10 respondents to improve wording and comprehensibility; these pilot respondents were not included in the main study sample or in any statistical analyses. This procedure was not a formal psychometric validation.

The questionnaire covered eligibility and consent; sociodemographic and health characteristics; current and previous supplement use; number and categories of products used; motivations and initiation of supplementation; professional involvement; information sources; self-reported safety-related supplementation behaviors; factual supplement safety knowledge; nutrition-related behaviors; and participation in ‘Profilaktyka 40+’. The complete questionnaire, including response options, is provided in Supplementary File S1, and the mapping from source questions to derived analytical variables is provided in Supplementary Table S1.

### 2.4. Measures and Variable Operationalization

#### 2.4.1. Sociodemographic and Health Variables

Age was analyzed as a continuous variable. Sex was analyzed as recorded in the questionnaire. Education was described using the original categories and dichotomized for multivariable modeling as higher education versus non-higher education to preserve degrees of freedom. Chronic disease and regular medication use were coded as yes/no variables from the corresponding questionnaire items. Body mass index (BMI) was calculated from cleaned self-reported height and weight after application of the documented data-cleaning rules. Free-text disease and medication entries were not retrospectively converted into new clinical categories without a prespecified coding dictionary.

#### 2.4.2. Supplement Use Status and Supplementation Patterns

Supplement use status was based on the questionnaire response categories regular current use, occasional current use, former use, and never use. Current users were respondents reporting regular or occasional current use; non-current users comprised former and never users. Among current users, regular use denoted use at least weekly, and occasional use was less than weekly. The number of simultaneously used supplement products was recorded as exactly 1, 2–3, 4–5, or >5. For this study, poly-supplementation was operationally defined a priori as concurrent use of ≥2 supplement products. This threshold was used as a descriptive exposure definition and was not intended to classify individual products or users as clinically ‘high risk’.

#### 2.4.3. Self-Reported Safety-Related Supplementation Behaviors

The study assessed several distinct self-reported behaviors with potential relevance to supplement safety rather than a single validated safety behavior construct. Dose adherence was coded as higher adherence when respondents reported always or often following the recommended dose and lower adherence when they reported sometimes or never doing so. Self-initiation identified respondents who reported starting supplementation on their own rather than following professional initiation. Professional oversight was coded as present when at least one healthcare professional option was selected as supervising or monitoring supplementation; respondents reporting no professional involvement were classified as not professionally overseen.

The primary behavioral outcome was proactive supplement safety information seeking among current supplement users. It was derived from the questionnaire item asking whether respondents checked potential supplement–medicine interactions or adverse effects and, if so, by which route. An affirmative response indicating physician consultation, pharmacy inquiry, or an internet/leaflet search was coded as proactive information seeking; reporting no information seeking was coded as absence of the outcome. Because the physician/pharmacy routes overlap conceptually with professional information-source use, additional sensitivity analyses decomposed this composite outcome into professional route and non-overlap internet/leaflet components (Section 2.6). These measures represent self-reported information-seeking behaviors and do not establish that an interaction, inappropriate dose, or adverse event actually occurred.

#### 2.4.4. Information Sources

Information-source variables were derived from the multiple-response item on sources used for supplement-related information. Professional information-source use was coded when a physician, pharmacist, and/or dietitian/nutrition specialist was selected. Digital information-source use was coded when internet articles/social media and/or online forums/groups were selected. These indicators were non-exclusive; respondents could use both professional and digital sources. A four-category descriptive variable (professional only, digital only, both, neither/other) was also derived. Because the survey was cross-sectional, the temporal ordering of information-source use and supplementation behaviors could not be established.

#### 2.4.5. Factual Supplement Safety Knowledge

The questionnaire contained six factual statements concerning supplement safety, answered as true, false, or ‘don’t know’. Before construction of the publication analysis score, the correctness and scoreability of the items were audited against authoritative evidence and the item wording. One compound item (P31.4) was excluded because it combined propositions that did not permit unambiguous binary correctness scoring. The resulting study-specific factual supplement safety knowledge score therefore comprised five items addressing supplement–medicine interactions, the misconception that ‘natural’ implies complete safety, regulatory differences between supplements and medicines, possible effects on regularly used medicines, and potential harm from exceeding recommended doses. Correct responses received one point and incorrect or ‘don’t know’ responses zero points; missing responses were not coded as incorrect. The score ranged from 0 to 5 and was calculated only when all five scoreable items were answered.

Additional measurement analyses were undertaken to characterize, rather than retrospectively validate, the five-item score. Internal consistency and item-level performance were examined, and sensitivity analyses evaluated whether conclusions depended on the continuous-score specification. These analyses are reported in the Supplementary Materials. The score is therefore described throughout as a study-specific factual knowledge measure and not as a formally validated psychometric scale.

#### 2.4.6. ‘Profilaktyka 40+’ Variables

Participation in the Polish ‘Profilaktyka 40+’ preventive-health program was recorded as yes/no. Follow-up questions among program participants assessed reasons for participation, receipt of nutrition or supplementation advice, self-reported lifestyle changes after participation, changes in supplementation, and perceived influence of program results on supplementation decisions. These follow-up items were structurally not applicable to nonparticipants. Analyses involving these variables were prespecified as exploratory and are reported primarily in the Supplementary Materials.

### 2.5. Data Quality Assurance, Missing Data, and Analytical Populations

Source data underwent sequential record, variable, logic, missing-data, outlier, coding, and final-freeze audits. Raw responses were preserved; structural missingness created by questionnaire routing was distinguished from item nonresponse; anthropometric entries were normalized according to documented rules; and multiple-response fields were regenerated and validated record by record. The corrected frozen dataset contained 150 unique analytical records with no unresolved blocking data quality issues. No outcome or covariate values were imputed. For paper questionnaires, transcription into the electronic database was performed by B.W. and independently verified by A.B. against the source paper forms before the analytical freeze.

The full cohort comprised 150 participants, including 113 current supplement users and 37 non-current users. Analyses restricted to current users used variable-specific denominators according to item availability. The five-item knowledge score was available for 103 current users, and the primary multivariable model included 102 complete cases because one additional participant lacked data required for that model. The original exploratory latent-class analysis included 108 current users with complete data for all seven indicators. ‘Profilaktyka 40+’ follow-up analyses were restricted to the 70 program participants where applicable. Exact denominators are reported for descriptive and inferential results, and the participant-flow diagram summarizes the principal analytical populations.

### 2.6. Statistical Analysis

Analyses were conducted according to a statistical analysis plan finalized before formal hypothesis testing. Descriptive statistics are reported as mean ± standard deviation or median where appropriate for continuous variables and as n/N (%) for categorical variables. Two-sided tests were used. The primary inferential analysis was a multivariable logistic regression among current supplement users with proactive supplement safety information seeking as the outcome and the five-item factual knowledge score as the exposure of interest. Age, sex, higher education, and regular medication use were included as prespecified covariates because they represented basic demographic and health factors plausibly related to supplement use, access to health information, or opportunities for supplement–medicine co-use; covariates were not selected or removed on the basis of statistical significance.

The primary complete-case model included 102 participants (68 with and 34 without the outcome). Multicollinearity was assessed using variance-inflation factors; values in the primary model were low (maximum VIF 1.41). Prespecified sensitivity analyses evaluated alternative analytical populations, categorical representations of knowledge, substitution of chronic disease for regular medication use, and additional adjustment for professional or digital information-source use. Additional analyses decomposed the primary outcome to assess measurement overlap between the professional-source exposure and the physician/pharmacy component of the composite information-seeking outcome. These non-overlap analyses were explicitly treated as additional sensitivity analyses rather than as replacements for the prespecified primary analysis.

Secondary logistic models evaluated associations involving professional oversight, self-initiation, poly-supplementation, dose adherence, and professional or digital information-source use. Models of professional and digital sources included both source indicators simultaneously together with the prespecified demographic and health covariates. When sparse events made conventional maximum-likelihood logistic regression unreliable, bias-reduced Firth logistic regression was used as prespecified. Benjamini–Hochberg false discovery rate correction was applied within prespecified families of secondary tests; both raw *p* values and adjusted q values are reported where applicable. Effect estimates are presented with 95% confidence intervals and interpreted according to magnitude, direction, and precision rather than statistical significance alone.

Exploratory analyses included Fisher exact tests and adjusted models for ‘Profilaktyka 40+’ outcomes and Bernoulli latent-class analysis (LCA) of seven binary behavioral indicators. One- to four-class solutions were compared using Bayesian information criterion (BIC), Akaike information criterion (AIC), relative entropy, class size, and posterior classification diagnostics. Because the LCA sample was modest and robustness analyses showed sensitivity to indicator specification, LCA findings are retained only as exploratory, hypothesis-generating results in the Supplementary Materials and are not used to support the principal conclusions.

All publication-grade statistical analyses were performed in Python 3.13.5 using pandas 2.2.3, NumPy 2.3.5, SciPy 1.17.0, and statsmodels 0.14.6. A two-sided *p* < 0.05 criterion was used for the primary model; FDR-adjusted q values are reported for prespecified families of secondary analyses.

### 2.7. Use of Generative Artificial Intelligence

During preparation and revision of the manuscript, the authors used ChatGPT (OpenAI, GPT-5.6) to assist with manuscript drafting and language editing and with generation/checking of analysis code. The authors independently reviewed the generated text, analytical outputs, and interpretations against the frozen study dataset and source documentation and take full responsibility for the final content. Generative AI was not used for participant recruitment, questionnaire administration, or collection of the source data.

## 3. Results

### 3.1. Participant Flow and Characteristics

**Figure 1:**
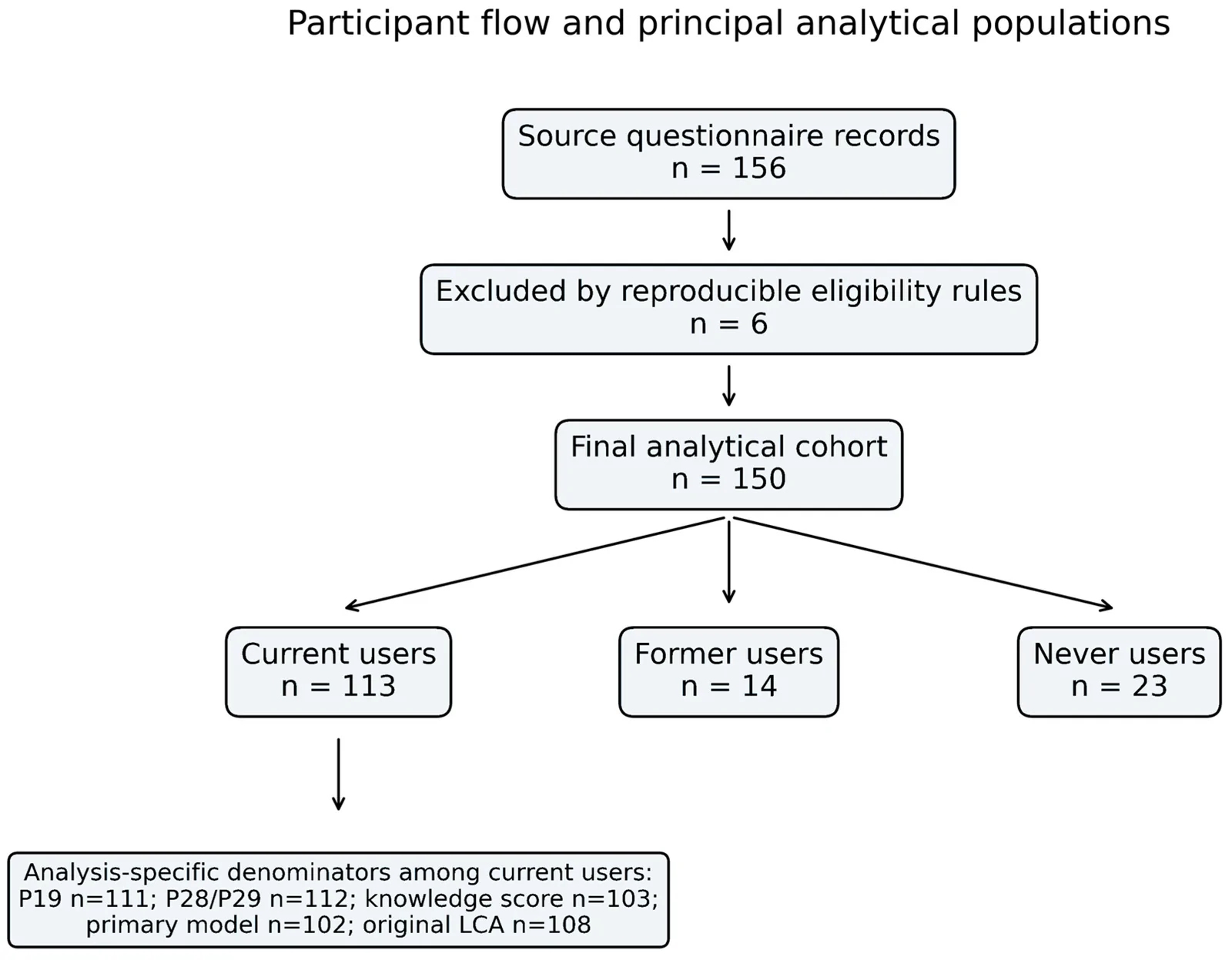
Participant flow and principal analytical populations.

The source dataset contained 156 response records. Six records did not meet the age-eligibility criterion, yielding a final analytical cohort of 150 adults. Of these, 113/150 (75.3%) were current dietary supplement users and 37/150 (24.7%) were former or never users. The mean age of the analytical cohort was 51.2 ± 10.1 years, 114/150 (76.0%) were women, 68/150 (45.3%) reported chronic disease, and 71/150 (47.3%) reported regular medication use. Participant characteristics according to current supplement use are shown in Table 1, and the principal analytical populations are summarized in Figure 1.

### 3.2. Supplement Use and Self-Reported Safety-Related Supplementation Behaviors

Among current users, 83/113 (73.5%) reported regular use. Among 111 current users with supplement count data, 25/111 (22.5%) reported using 1 product, 65/111 (58.6%) 2–3 products, 14/111 (12.6%) 4–5 products, and 7/111 (6.3%) >5 products simultaneously; thus, 86/111 (77.5%) used ≥2 products. Because P19 recorded product count in ordinal categories, including the open-ended >5 category, an arithmetic mean number of products cannot be estimated without imposing arbitrary numeric values. Dose adherence (always/often following the recommended dose) was reported by 101/113 (89.4%), whereas 67/113 (59.3%) reported self-initiated supplementation. Professional oversight was reported by 38/112 (33.9%), while 64/112 (57.1%) explicitly reported self-directed supplementation without professional oversight. Proactive supplement safety information seeking was reported by 77/112 (68.8%). Professional information sources were used by 58/112 (51.8%) and digital sources by 71/112 (63.4%) (Table 2). Detailed supplement category frequencies and thematic product clusters are reported in the Supplementary Materials rather than duplicated in the main text.

### 3.3. Factual Supplement Safety Knowledge and the Primary Outcome

A complete five-item factual supplement safety knowledge score was available for 103 current users. The mean score was 3.52 ± 1.49 of 5, the median was 4, and 38/103 (36.9%) answered all five scoreable items correctly. Item-level performance and additional measurement analyses are reported in the Supplementary Materials. The prespecified primary complete-case model included 102 current users, of whom 68 had and 34 did not have the primary outcome. Each one-point increase in the knowledge score was associated with an adjusted odds ratio (aOR) of 0.93 for proactive supplement safety information seeking (95% CI 0.69–1.25; *p* = 0.641) after adjustment for age, sex, higher education, and regular medication use (Table 3). The confidence interval was compatible with associations of modest magnitude in either direction and did not support a precise independent association in this pilot sample. The primary conclusion was unchanged across the prespecified sensitivity analyses reported in the Supplementary Materials.

### 3.4. Information Sources and Safety-Related Supplementation Behaviors

**Figure 2:**
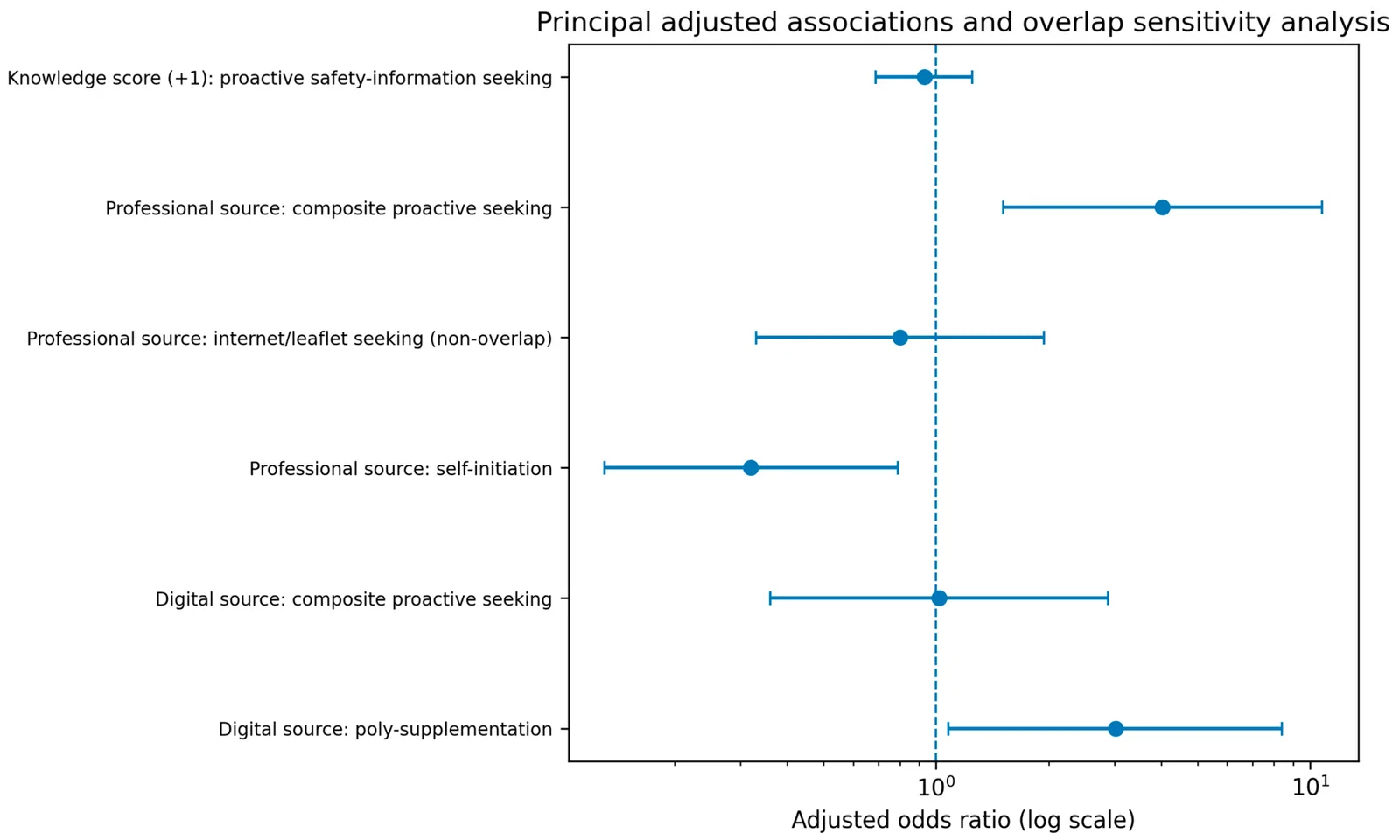
Principal adjusted associations and measurement-overlap sensitivity analysis. Points show adjusted odds ratios and horizontal lines show 95% confidence intervals; the vertical reference line denotes an odds ratio of 1. The non-overlap sensitivity outcome excludes the physician/pharmacy route from the composite proactive supplement safety information-seeking outcome.

Among 112 current users with information-source data, 29/112 (25.9%) used professional sources only, 42/112 (37.5%) digital sources only, 29/112 (25.9%) both, and 12/112 (10.7%) neither source group. In the original adjusted model using the composite proactive information-seeking outcome, professional information-source use was associated with higher odds of the outcome (aOR 4.03, 95% CI 1.51–10.77; raw *p* = 0.0054; BH q = 0.0326), whereas digital-source use was not (aOR 1.02, 95% CI 0.36–2.88; *p* = 0.9671). However, the non-overlap sensitivity analysis, which restricted the outcome to internet/leaflet safety information seeking and thereby removed the physician/pharmacy route shared conceptually with professional-source exposure, did not show an independent association with professional-source use (aOR 0.80, 95% CI 0.33–1.94; *p* = 0.6269) or digital-source use (aOR 1.39, 95% CI 0.54–3.63; *p* = 0.4958) (Table 4; Figure 2). Thus, the original professional-source association with the composite outcome should be interpreted in light of measurement overlap.

Professional information-source use was also associated with lower odds of self-initiated supplementation (aOR 0.32, 95% CI 0.13–0.79; raw *p* = 0.0139; BH q = 0.0418). The association between digital-source use and poly-supplementation was positive but did not remain below the prespecified FDR threshold (aOR 3.02, 95% CI 1.08–8.41; raw *p* = 0.0344; BH q = 0.0688). Complete secondary models and additional sensitivity analyses are provided in the Supplementary Materials.

### 3.5. Exploratory Analyses

Exploratory analyses of participation in the ‘Profilaktyka 40+’ program, supplement category combinations, and latent behavioral profiles are reported in the Supplementary Materials. Among the 70 program participants, 46/70 (65.7%) reported receiving no nutrition or supplementation advice, 20/70 (28.6%) brief guidance, and 4/70 (5.7%) detailed information. The original seven-indicator latent-class analysis included 108 current users; although a two-class solution had the lowest BIC, robustness analyses indicated sensitivity to indicator specification. These findings are therefore retained as hypothesis-generating exploratory results and are not used to support the principal conclusions.

## 4. Discussion

### 4.1. Principal Findings

This pilot cross-sectional study examined whether a study-specific measure of factual supplement safety knowledge and the use of professional or digital information sources were associated with self-reported supplementation behaviors among adults aged ≥40 years in a convenience sample from Poland. Three findings merit emphasis. First, the five-item factual knowledge score was not independently associated with proactive supplement safety information seeking in the primary model (aOR 0.93, 95% CI 0.69–1.25), and the estimate remained similar across prespecified sensitivity analyses. The confidence interval, however, does not rule out an association and remains compatible with modest associations in either direction. Second, professional information-source use was associated with the original composite information-seeking outcome and with lower odds of self-initiation. Third, the route decomposition analysis materially qualified the first of these professional-source findings: when the outcome was restricted to internet/leaflet safety information seeking and the physician/pharmacy route was removed, professional-source use was no longer independently associated with the outcome. The original fourfold association therefore cannot be interpreted as straightforward evidence that professional-source use promotes safer supplementation.

These findings refine, rather than overturn, the study’s original interpretation. They suggest that factual knowledge, information-source use, and self-reported safety-related behaviors are distinct, although potentially related, aspects of supplement decision-making. At the same time, the cross-sectional design, measurement overlap in the original composite outcome, and potential residual confounding by healthcare engagement or health literacy prevent conclusions about directionality or causal effects of professional contact.

### 4.2. Supplement Use, Poly-Supplementation, and the Safety Concern

Current supplement use was reported by 75.3% of the analytical cohort. This value should be interpreted only as a characteristic of this convenience sample and not as a prevalence estimate for Polish adults aged ≥40 years. Estimates from representative and other population-based studies differ substantially according to age, sampling frame, definitions, and ascertainment methods [1,4,15,16,17,18]. The high proportion in the present cohort may also reflect self-selection of individuals with greater interest in health or supplementation.

Among current users with supplement count data, 77.5% used at least two supplement products concurrently. This finding is relevant because concurrent use increases the complexity of potential combined exposures, particularly when supplements are used alongside medicines. However, poly-supplementation is not synonymous with unsafe supplementation, and the present study did not verify brand-level products, ingredient doses, contraindications, or actual supplement–medicine interactions. Reviews have documented clinically relevant interactions involving some herbs and dietary supplements [5,6], and concurrent use of medicines, herbs, and nutritional supplements can create complex exposure patterns in older adults [7,19,20]. These data support attention to product-specific assessment, but they do not justify treating herbal supplements as a uniformly ‘riskier’ category.

This distinction is important when considering whether the findings would be reproduced in a larger sample containing more users of products with greater interaction potential. The present dataset cannot answer that question because it was not designed or powered for product-level risk stratification. The sample included vitamins, minerals, omega-3 fatty acids, collagen/joint preparations, herbal products, and other categories, but product composition and dose were not independently verified. Accordingly, the practical significance of the present pilot study lies in characterizing information-seeking and supplement-management behaviors, not in estimating clinical risk associated with particular supplement categories. Future studies should prospectively record exact products, ingredients, doses, duration, concomitant medicines, and relevant health conditions so that interaction potential can be assessed at the individual level.

### 4.3. Factual Knowledge and Self-Reported Safety-Related Behavior

The primary hypothesis that higher factual supplement safety knowledge would be associated with greater proactive supplement safety information seeking was not supported in this sample. This result should not be simplified to the conclusion that knowledge ‘does not matter’. The five-item score was study-specific, had limited internal consistency in the measurement analysis, and was not a validated measure of supplement literacy or health literacy. It captured a narrow set of factual statements and therefore may not represent the broader competencies required to recognize personal risk, evaluate sources, or act on information.

Previous research also indicates that supplement-related knowledge operates within a broader behavioral context. Karbownik et al. found that knowledge about dietary supplements was associated with beliefs, attitudes, and information-related characteristics among Polish Internet users [9]. Bayır et al. examined knowledge together with health consciousness, safety concerns, attitudes, subjective norms, and behavioral intentions [10]. Other cross-sectional studies have likewise reported imperfect correspondence between knowledge and supplement-related practices [11,21,22]. Although these studies used different instruments and outcomes, collectively they support treating factual knowledge and behavior as distinct constructs rather than assuming a direct knowledge-to-action pathway.

The precision of the primary estimate is also important. An aOR of 0.93 with a 95% CI of 0.69–1.25 is compatible with both a modest inverse and a modest positive association. With the present sample size, the study therefore did not identify a clear independent association; it does not demonstrate equivalence or prove the absence of an effect. Larger studies using validated measurement instruments are needed to estimate the magnitude of any knowledge–behavior association more precisely.

### 4.4. Professional and Digital Information Pathways

In the prespecified analysis, professional information-source use was associated with higher odds of the composite proactive information-seeking outcome and lower odds of self-initiated supplementation. The latter association is conceptually plausible because consultation with physicians, pharmacists, or dietitians represents a form of professional involvement in supplement decisions. Previous studies have documented the role of healthcare professionals within a broader information environment in which consumers also use multiple non-professional sources [12,23,24]. However, professional involvement cannot be assumed to occur routinely. In a recent survey of German general practitioners, more than one-third reported rarely or never addressing dietary supplements during periodic health examinations [25]. Challenges have also been identified in the documentation of supplement use within healthcare systems and in communication about supplement use, with implications for medication reconciliation and patient safety [26].

The association with the composite proactive information-seeking outcome requires substantially greater caution. The exposure classified use of professional information sources, whereas the original outcome counted physician consultation and pharmacy inquiry among the routes constituting proactive information seeking. The non-overlap sensitivity analysis removed those professional routes from the outcome and retained only internet/leaflet safety-information seeking. With this outcome definition, professional-source use was not independently associated with the outcome (aOR 0.80, 95% CI 0.33–1.94). This finding indicates that conceptual measurement overlap contributed to, or may have substantially influenced, the original association.

Residual confounding is another plausible explanation. Individuals who are more safety-oriented, have greater health literacy, use regular medicines, or have more frequent healthcare contact may be more likely both to consult professionals and to report safety-information seeking. Because these characteristics were not comprehensively measured, adjustment for age, sex, education, and medication use cannot eliminate this possibility. Reverse directionality is also possible: a perceived supplement-related concern may prompt professional consultation rather than professional contact preceding the behavior. The present data therefore support an association between professional engagement and some self-reported supplementation behaviors, but they do not establish that professional engagement caused safer behavior.

Digital information-source use was common but was not independently associated with the primary composite outcome. A positive association with poly-supplementation did not meet the prespecified FDR-adjusted threshold. Digital-source use should therefore not be characterized as inherently unsafe or as an independent driver of poly-supplementation on the basis of these data. Digital sources vary greatly in quality, and future work should distinguish source credibility, platform type, content exposure, and the ability to appraise information rather than treating digital use as a single category [27]. A systematic review of online nutrition-related information found substantial variability in both quality and accuracy across websites and social-media content [28], while an international survey showed that frequently consulted information sources were not necessarily those perceived as most trustworthy [13].

### 4.5. What This Pilot Study Adds

The principal contribution of this study is the integration of factual knowledge, information-source pathways, professional involvement, supplement use patterns, and specific self-reported safety-related behaviors within one analytical framework. Much previous research has focused on prevalence, motivations, knowledge, attitudes, or information sources, although more recent work has begun to integrate several of these dimensions when examining dietary supplement users [1,9,12,14,15,16,17,18,21]. In this pilot sample, a narrow factual knowledge score did not clearly distinguish participants who reported proactive supplement safety information seeking from those who did not, while associations involving professional involvement and self-initiation warrant further study. Importantly, the overlap sensitivity analysis shows that the observed associations depend in part on how exposures and outcomes are defined. These findings also highlight the importance of careful outcome definition in future questionnaire-based research on supplement safety.

### 4.6. Exploratory Findings

The analyses concerning the ‘Profilaktyka 40+’ program and latent behavioral classes were exploratory and are presented primarily in the Supplementary Materials. Program participants who reported receiving nutrition- or supplementation-related advice also more often reported subsequent changes; however, self-selection, retrospective reporting, and the absence of temporal verification preclude causal interpretation. Similarly, the original two-class latent-class solution was interpretable, but the LCA sample was modest (n = 108), relative entropy was acceptable rather than high, and robustness analyses showed sensitivity to indicator specification. The classes should therefore be regarded as hypothesis-generating patterns rather than stable consumer phenotypes.

### 4.7. Strengths and Limitations

Strengths of the study include reconstruction and audit of the source dataset, explicit analytical population definitions, a statistical analysis plan finalized before formal hypothesis testing, separation of primary, secondary, and exploratory analyses, complete-case and sensitivity analyses, reporting of confidence intervals, and FDR correction for prespecified families of secondary tests. Targeted measurement analyses also examined the knowledge score and the P28/P29 outcome exposure structure, allowing limitations in the original operationalization to be identified and reported transparently.

The limitations are substantial and define the scope of inference. First, this was a pilot study using convenience sampling, with 76% women and a high proportion of current supplement users. The cohort is therefore not representative, and neither prevalence estimates nor behavioral associations should be generalized to Polish adults aged ≥40 years without replication. Second, the modest sample size limited precision and model complexity. This is evident in several wide confidence intervals and is particularly relevant to subgroup, product pattern, and latent-class analyses.

Third, the cross-sectional design prevents determination of temporal sequence and causality. Professional-source use may precede, follow, or occur as part of the same information-seeking episode as the reported behavior. Residual confounding by unmeasured health engagement, health literacy, healthcare contact, risk perception, or other individual characteristics is probable. Fourth, the central measures were self-reported and are susceptible to recall and social desirability bias. The primary outcome was binary and combined interaction and adverse-effect information seeking; it measured reported checking behavior rather than the occurrence or prevention of adverse events.

Fifth, the questionnaire was author-developed and underwent content review and pilot testing but not formal psychometric validation. The five-item knowledge score was study-specific and showed limited internal consistency; it should not be interpreted as a validated scale of supplement safety literacy. Sixth, although the study recorded supplement categories and number of products, it did not independently verify exact formulations, ingredient doses, duration of use, medication names, or actual interactions. It therefore cannot determine whether participants used products with greater interaction potential or whether poly-supplementation represented clinically unsafe use. This limitation directly constrains the practical interpretation of the findings and is a major reason for framing the study as pilot research. Finally, the ‘Profilaktyka 40+’ and LCA analyses were exploratory and require replication in larger independent samples.

### 4.8. Future Research and Practical Applications

The findings support several directions for future research rather than immediate population-level recommendations. Larger, more diverse samples should combine validated measures of supplement knowledge or health literacy with detailed product-level exposure data, including exact ingredients, doses, duration, concomitant medicines, and relevant health conditions. Prospective designs would help establish whether professional consultation precedes changes in supplement management behavior and would reduce uncertainty about reverse directionality.

From a practical perspective, the results are consistent with the value of routinely asking patients about dietary supplement use as part of a complete medication and health product history, particularly when several supplements and prescription medicines are used concurrently. Such information may help healthcare professionals identify complex patterns of combined exposure, discuss potential supplement–medicine interactions, and direct patients toward reliable sources of safety information. Recent studies have identified both opportunities and barriers to such professional involvement, including incomplete discussion and documentation of supplement use in healthcare settings [25,26]. Community pharmacists are particularly accessible potential sources of supplement counselling, although studies indicate variability in knowledge and preparedness for this role [29,30]. This implication follows from the complexity of combined exposures and the established literature on potential supplement–medicine interactions [5,6,7,19,20], not from evidence in the present study that professional engagement itself prevents harm. Future intervention studies should test whether structured supplement reconciliation, pharmacist or dietitian review, or high-quality digital decision support improves verified safety outcomes rather than self-reported information seeking alone.

## 5. Conclusions

In this pilot cross-sectional convenience sample of adults aged ≥40 years, the study-specific five-item factual supplement safety knowledge score showed no clear independent association with proactive supplement safety information seeking. The estimate was imprecise (aOR 0.93, 95% CI 0.69–1.25) and should not be interpreted as evidence that supplement safety knowledge is unimportant. Professional information-source use was associated with the original composite information-seeking outcome and with lower odds of self-initiated supplementation; however, the former association was materially qualified by the non-overlap sensitivity analysis, indicating that conceptual overlap between exposure and outcome contributed to the original finding.

Accordingly, these results do not establish that professional engagement causes safer supplementation, nor do they permit conclusions about the clinical safety of particular supplement categories. The study did not verify exact products, ingredient doses, supplement–medicine interactions, or adverse events, and its convenience sample was not population-representative. The findings should therefore be regarded as pilot evidence supporting larger prospective studies that combine validated measures of knowledge and health literacy with product-level supplement and medication data and objectively verifiable safety outcomes. For clinicians and researchers, the results also underscore the importance of defining supplement safety behaviors and information-source exposures so that conceptually overlapping measures are not interpreted as independent effects.

## Figures and Tables

**Table 1:** Characteristics of the analytical cohort according to current dietary supplement use.

Characteristic	Overall (n = 150)	Current Users (n = 113)	Non-Current Users (n = 37)
Age, years	51.2 ± 10.1	51.4 ± 9.9	50.3 ± 10.8
BMI, kg/m^2^	26.9 ± 4.4	26.5 ± 4.2	27.8 ± 5.0
Female sex, n (%)	114 (76.0)	85 (75.2)	29 (78.4)
Higher education, n (%)	70 (46.7)	52 (46.0)	18 (48.6)
Chronic disease, n (%)	68 (45.3)	51 (45.1)	17 (45.9)
Regular medication use, n (%)	71 (47.3)	55 (48.7)	16 (43.2)
Participation in ‘Profilaktyka 40+’, n (%)	70 (46.7)	60 (53.1)	10 (27.0)

Data are mean ± SD for continuous variables and n (%) for categorical variables. Table 1 is descriptive; no hypothesis-testing *p* values are presented.

**Table 2:** Supplement use patterns and self-reported safety-related supplementation behaviors among current users.

Domain	Characteristic	n/N	%
Supplement use pattern	Regular current use	83/113	73.5
Supplement use pattern	Poly-supplementation (≥2 simultaneous products)	86/111	77.5
Safety-related behavior	Dose adherence (always/often followed recommended dose)	101/113	89.4
Safety-related behavior	Self-initiated supplementation	67/113	59.3
Safety-related behavior	Professional oversight	38/112	33.9
Safety-related behavior	Self-directed/no professional oversight	64/112	57.1
Safety-related behavior	Proactive supplement safety information seeking	77/112	68.8
Information source	Any professional information source	58/112	51.8
Information source	Any digital information source	71/112	63.4

Variable-specific denominators reflect item availability. Poly-supplementation was operationally defined for this study as concurrent use of ≥2 supplement products.

**Table 3:** Primary logistic regression model for proactive supplement safety information seeking.

Predictor	Crude OR	95% CI	*p*	Adjusted OR	95% CI	*p*
Knowledge score (+1 point)	0.90	0.68–1.20	0.481	0.93	0.69–1.25	0.641
Age (+10 years)	1.08	0.72–1.64	0.701	1.00	0.58–1.72	0.994
Male sex	0.33	0.14–0.81	0.016	0.38	0.14–1.05	0.062
Higher education	1.74	0.77–3.94	0.186	1.36	0.49–3.79	0.558
Regular medication use	1.71	0.76–3.84	0.196	1.69	0.68–4.19	0.259

Primary adjusted model: n = 102; 68 events and 34 non-events. The adjusted model included the knowledge score, age, sex, higher education, and regular medication use. Age is expressed per 10-year increase. OR, odds ratio; CI, confidence interval.

**Table 4:** Associations of professional and digital information-source use with supplementation behaviors, including overlap sensitivity analysis.

Outcome	Exposure	n	Events	aOR	95% CI	Raw *p*	BH q
Composite proactive supplement safety information seeking	Professional information source	102	68/102	4.03	1.51–10.77	0.0054	0.0326
Composite proactive supplement safety information seeking	Digital information source	102	68/102	1.02	0.36–2.88	0.9671	0.9671
Internet/leaflet safety-information seeking (non-overlap sensitivity)	Professional information source	102	39/102	0.80	0.33–1.94	0.6269	—
Internet/leaflet safety-information seeking (non-overlap sensitivity)	Digital information source	102	39/102	1.39	0.54–3.63	0.4958	—
Self-initiated supplementation	Professional information source	103	65/103	0.32	0.13–0.79	0.0139	0.0418
Self-initiated supplementation	Digital information source	103	65/103	1.50	0.59–3.82	0.3923	0.4708
Poly-supplementation	Professional information source	110	85/110	2.05	0.74–5.66	0.1662	0.2493
Poly-supplementation	Digital information source	110	85/110	3.02	1.08–8.41	0.0344	0.0688

Professional and digital source indicators were included simultaneously. Composite proactive-seeking and self-initiation models additionally included the five-item knowledge score, age, sex, higher education, and regular medication use; the poly-supplementation model used the corresponding prespecified covariate set. The internet/leaflet outcome is an additional non-overlap sensitivity analysis and was not part of the prespecified primary analysis. aOR, adjusted odds ratio; CI, confidence interval; BH q, Benjamini–Hochberg false-discovery-rate-adjusted *p* value.

## Data Availability

The raw data supporting the conclusions of this article will be made available by the authors on request.

## Supplementary Materials

The following supporting information can be downloaded at: https://www.mdpi.com/article/10.3390/nu18172921/s1, Table S1. Questionnaire-to-variable measurement dictionary; Table S2A. Reported dietary supplement categories among current users; Table S2A-1. Number of dietary supplement products used simultaneously among current users (P19); Table S2B. Ten most frequent supplement-category pairs; Table S2C. Clinically relevant medication/poly-supplementation subgroup; Table S3. Item performance and exploratory measurement properties of the five-item factual supplement-safety knowledge score; Table S4. Primary-model and revision-stage sensitivity analyses; Table S5A. Complete secondary multivariable model results; Table S5B. Model adequacy and diagnostics; Table S6. Analytical populations, variable availability, and missing-data handling; Table S7. Descriptive information-source profiles among current users; Table S8A. Exploratory ‘Profilaktyka 40+’ programme responses; Table S8B. Exploratory associations with receiving ‘Profilaktyka 40+’ nutrition/supplement advice; Table S8C. Exploratory association between ‘Profilaktyka 40+’ programme participation and current supplement use; Table S9. Latent-class model selection and revision-stage indicator-specification robustness; Figure S1. Item-level factual supplement-safety knowledge.; Figure S2. Reported dietary supplement categories among current users.; Figure S3. Exploratory associations with receiving ‘Profilaktyka 40+’ nutrition/supplement advice.; Figure S4. Original exploratory two-class behavioural solution; Supplementary File S1. Complete questionnaire—English reporting translation.
