# Supplementary material for: Supplement Safety Knowledge, Information Sources, and Self-Reported Safety-Related Behaviors Among Adults Aged 40 Years and Older: A Pilot Study
Source: Nutrients. 2026 Sep 7;18(17):2921. doi: 10.3390/nu18172921 (PMC13567739; doi:10.3390/nu18172921)
Supplement: Supplementary file 1 [file nutrients-18-02921-s001.zip › nutrients-4531400-supplementary.pdf]

Supplement-Safety Knowledge, Information Sources, and Self-Reported Safety-Related Behaviors among Adults Aged 40 Years and Older: A Pilot Study

Beata Walczyk and Agnieszka Białek

Revision-stage supplementary package. The analyses below preserve the frozen SUPPLEMENT-GOLD Dataset v1.0.1 and distinguish analyses specified before formal hypothesis testing from reviewer-driven revision-stage sensitivity and measurement audits. These supplementary analyses expand reporting transparency and do not replace the prespecified primary analysis. The administered questionnaire contained 46 numbered questions; the 10 pilot-test respondents used for wording/comprehensibility assessment were not included in the main study sample or statistical analyses.

Supplementary Table S1. Questionnaire-to-variable measurement dictionary

Construct	Source item	Original response structure	Derived coding	Applicable population
Current supplement use	P18	Regular or occasional current use	1=current; 0=former/never	Full cohort
Regular current use	P18	Regular (≥weekly) vs occasional (<weekly)	Binary among current users	Current users
Poly-supplementation	P19	1; 2–3; 4–5; >5 simultaneously	1=≥2 products; 0=1 product	Current users
Dose adherence	P25	Always; often; sometimes; never	1=always/often; 0=sometimes/never	Current users
Professional oversight	P26	Healthcare-professional and non-professional oversight options	1=any physician/primary-care-pharmacist/dietitian/other medical professional; 0=no professional oversight	Current users
Self-initiation	P23	Multiple possible initiators	1=self-decision selected; 0=otherwise	Current users
Proactive supplement-safety information seeking	P28	Physician consultation; pharmacy enquiry; internet/leaflet search; no information sought	1=any active route; 0=no information sought	Current users
Professional information source	P29	Physician; pharmacist; dietitian; other sources	1=physician and/or pharmacist and/or dietitian	Current users
Digital information source	P29	Internet/social media; online forums/groups; other sources	1=internet/social media and/or online forums/groups	Current users
Five-item factual supplement-safety knowledge score	P31	Six factual statements; T/F/Don’t know;	0–5; correct=1; incorrect/Don’t know=0;	Current/supplement-experienced as specified

		one compound item excluded from scoreability audit	complete five-item responses required	
'Profilaktyka 40+' participation	P41–P46	Participation and programme-specific follow-up items	Exploratory variables; P42–P46 structurally unavailable for nonparticipants	Programme participants

The complete English reporting translation of the questionnaire is provided in Appendix S1. The original questionnaire was administered in Polish; the English version is provided for transparency and was not independently validated as a translated instrument.

Supplementary Table S2A. Reported dietary supplement categories among current users

Category	Presentation group	n/N	%
Vitamin D	Vitamins/minerals	91/112	81.2
Vitamin C	Vitamins/minerals	58/112	51.8
Calcium/magnesium/potassium preparations	Vitamins/minerals	45/112	40.2
B-group vitamins	Vitamins/minerals	35/112	31.2
Omega-3 fatty acids	Fatty acids	31/112	27.7
Collagen/joint preparations	Specialty products	28/112	25.0
Herbal products	Botanicals	27/112	24.1
Multivitamin preparations	Vitamins/minerals	26/112	23.2
Probiotics/prebiotics	Microbiome-related	22/112	19.6
Antioxidants (e.g., coenzyme Q10)	Other bioactive products	12/112	10.7
Protein preparations	Protein/sports	5/112	4.5

Presentation groups are descriptive organizational labels only and are not risk categories. Broad questionnaire categories do not permit valid retrospective classification of individual products as “higher-risk” or “lower-risk”.

Supplementary Table S2A-1. Number of dietary supplement products used simultaneously among current users (P19)

P19 response category	n/N	%
1 product	25/111	22.5
2–3 products	65/111	58.6
4–5 products	14/111	12.6
>5 products	7/111	6.3
≥2 products	86/111	77.5

P19 recorded supplement count in ordinal categories (1, 2–3, 4–5, >5). Therefore, an arithmetic mean cannot be calculated without assigning arbitrary numeric values to grouped categories, particularly the open-ended >5 category. Two of 113 current users had missing P19 data; percentages use N=111.

Supplementary Table S2B. Ten most frequent supplement-category pairs

Supplement-category pair	n	% of 112 coded current users
**Vitamin D + Vitamin C**	51	45.5
**Vitamin D + Calcium/magnesium/potassium**	34	30.4
**Vitamin D + B-group vitamins**	32	28.6
**Vitamin D + Omega-3**	29	25.9

**Vitamin D + Collagen/joint**	23	20.5
**Vitamin D + Herbal products**	22	19.6
**Vitamin C + Calcium/magnesium/potassium**	21	18.8
**Vitamin C + B-group vitamins**	20	17.9
**Vitamin D + Multivitamin**	20	17.9
**Vitamin C + Multivitamin**	19	17.0

Pair frequencies are descriptive; participants could report multiple categories and multiple pairs.

Supplementary Table S2C. Clinically relevant medication/poly-supplementation subgroup

Characteristic	n/N	%
**Current users reporting regular medication**	55/113	48.7
**Poly-supplementation among medication users**	38/53	71.7
**Medication + poly-supplementation among all current users**	38/113	33.6
**Professional oversight in medication + poly subgroup**	18/37	48.6
**No professional oversight in medication + poly subgroup**	19/37	51.4
**Proactive safety-information seeking in medication + poly subgroup**	31/38	81.6

These data characterize regimen complexity and professional involvement; they do not establish clinically verified supplement–medicine interactions or individual risk.

Supplementary Table S3. Item performance and exploratory measurement properties of the five-item factual supplement-safety knowledge score

Scoreable item	Correct/answered	Correct (%)	Missing n	Corrected item–total r	α if item deleted
Supplements can interact with medicines	76/110	69.1	3	0.51	0.63
“Natural” does not mean completely safe	56/111	50.5	2	0.56	0.60
Supplements are less rigorously regulated than medicines	67/108	62.0	5	0.42	0.67
Supplements can affect regularly used medicines	89/109	81.7	4	0.49	0.64
Exceeding the recommended dose may be harmful	96/111	86.5	2	0.32	0.70

Complete-case n=103 current users. Overall Cronbach α=0.70; KR-20=0.71. These revision-stage analyses indicate moderate internal coherence but do not constitute formal psychometric validation. The sixth questionnaire statement (P31.4) was excluded from scoring because its compound wording did not permit unambiguous binary correctness coding.

Supplementary Table S4. Primary-model and revision-stage sensitivity analyses

Analysis	n	Events	Focal term	OR/aOR	95% CI	p
**Primary main**	102	68	Knowledge score (+1)	0.93	0.69–1.25	0.641
**S2 supplement-experienced**	111	75	Knowledge score (+1)	0.91	0.69–1.21	0.533
**S3 knowledge=3 vs 0–2**	102	68	Knowledge=3 vs 0–2	0.46	0.11–1.90	0.286
**S3 knowledge=4–5 vs 0–2**	102	68	Knowledge=4–5 vs 0–2	0.73	0.25–2.13	0.563

**S4 chronic disease instead of medication**	102	68	Knowledge score (+1)	0.92	0.69–1.24	0.592
**S5 + professional info source**	102	68	Knowledge score (+1)	0.94	0.69–1.28	0.682
**S5 + digital info source**	102	68	Knowledge score (+1)	0.94	0.70–1.27	0.705
**Non-overlap: internet/leaflet outcome**	102	39	Professional information source	0.80	0.33–1.94	0.627
**Overlap-linked: physician/pharmacy outcome**	102	37	Professional information source	17.58	5.20–59.46	<0.0001

The non-overlap internet/leaflet analysis was added during revision to evaluate the reviewer-identified channel overlap between P28 and P29. The large association for the physician/pharmacy route demonstrates that the original professional-source/composite-outcome association is substantially channel-overlap-sensitive.

Supplementary Table S5A. Complete secondary multivariable model results

Model	n	Events	Predictor	aOR	95% CI	p	BH q
**Professional oversight**	103	32	Knowledge score (+1)	0.72	0.53–0.98	0.035	0.088
**Professional oversight**	103	32	Age (+10 y)	0.86	0.50–1.46	0.567	0.709
**Professional oversight**	103	32	Male	0.89	0.29–2.72	0.832	0.832
**Professional oversight**	103	32	Higher education	0.44	0.15–1.27	0.129	0.214
**Professional oversight**	103	32	Regular medication	3.52	1.34–9.26	0.011	0.054
**Poly-supplementation**	111	86	Age (+10 y)	0.77	0.47–1.24	0.283	0.719
**Poly-supplementation**	111	86	Male	0.81	0.27–2.46	0.714	0.719
**Poly-supplementation**	111	86	Higher education	0.81	0.28–2.38	0.708	0.719
**Poly-supplementation**	111	86	Regular medication	0.61	0.24–1.57	0.306	0.719
**Poly-supplementation**	111	86	Self-initiated	1.19	0.47–2.99	0.719	0.719
**Current supplement use**	150	113	Age (+10 y)	1.08	0.70–1.67	0.722	0.910
**Current supplement use**	150	113	Male	1.18	0.46–3.03	0.735	0.910
**Current supplement use**	150	113	Higher education	0.95	0.40–2.25	0.910	0.910
**Current supplement use**	150	113	Chronic disease	0.48	0.12–1.95	0.307	0.767
**Current supplement use**	150	113	Regular medication	2.19	0.53–8.96	0.278	0.767
**Regular vs occasional current use**	113	83	Age (+10 y)	0.85	0.53–1.36	0.485	0.485
**Regular vs occasional current use**	113	83	Male	0.63	0.23–1.71	0.364	0.485
**Regular vs occasional current use**	113	83	Higher education	2.47	0.87–7.05	0.090	0.332
**Regular vs occasional current use**	113	83	Regular medication	1.92	0.76–4.83	0.166	0.332

BH q values are reported for the pre-specified within-model secondary families. Exploratory findings are not promoted to confirmatory conclusions.

Supplementary Table S5B. Model adequacy and diagnostics

Model	n	Events	Non-events	Terms	Estimation	Convergence	VIF/diagnostic note

**Primary knowledge model**	102	68	34	5	Maximum-likelihood logistic	Converged	1.41
**Professional oversight**	103	32	71	5	Maximum-likelihood logistic	Converged	Not problematic
**Poly-supplementation**	111	86	25	5	Maximum-likelihood logistic	Converged	Not problematic
**Current supplement use**	150	113	37	5	Maximum-likelihood logistic	Converged	3.31 (moderate health-variable collinearity)
**Dose-adherence sparse outcome**	113	101	12	—	Firth logistic	Converged	—

No blocking convergence or separation problem was identified in the main maximum-likelihood models. Firth logistic regression was used for the sparse dose-adherence outcome.

Supplementary Table S6. Analytical populations, variable availability, and missing-data handling

Analysis/variable	Eligible population	Eligible N	Available N	Missing N	Reason for reduced denominator	Handling
**Full analytical cohort**	All eligible respondents	150	150	0	Eligibility-filtered cohort	No imputation
**Current supplement users**	P18 regular/occasional	113	113	0	Questionnaire classification	Not applicable
**Supplement-experienced**	Current + former users	127	127	0	Questionnaire classification	Not applicable
**Supplement count (P19)**	Current users	113	111	2	Item nonresponse	Available-case
**Dose adherence (P25)**	Current users	113	113	0	None	Available-case
**Professional oversight (P26)**	Current users	113	112	1	Item nonresponse	Available-case
**Safety-information seeking (P28)**	Current users	113	112	1	Item nonresponse	Available-case
**Information sources (P29)**	Current users	113	112	1	Item nonresponse	Available-case
**Five-item knowledge score**	Current users	113	103	10	Requires all five scoreable items	No imputation
**Primary multivariable model**	Current users	113	102	11	Complete required model variables	Complete-case
**LCA original indicator set**	Current users	113	108	5	Complete seven-indicator profile	Complete-case
**'Profilaktyka 40+' analyses**	Programme participants	70	70	0	P41 routing	Structural NA outside programme

Structural missingness due to questionnaire routing was distinguished from item nonresponse. Structural missing values and outcomes were not imputed; the five-item knowledge score required complete responses to all five scoreable items.

Supplementary Table S7. Descriptive information-source profiles among current users

Source profile	n	Mean knowledge	Proactive seeking (%)	Professional oversight (%)	Poly-supplementation (%)	Self-initiation (%)
**Professional only**	29	3.19	79.3	72.4	69.0	41.4
**Digital only**	42	3.64	57.1	7.1	80.0	76.2
**Both**	29	3.77	82.1	50.0	89.7	48.3
**Neither/other**	12	3.33	50.0	0.0	58.3	66.7

Professional and digital sources were non-exclusive; the four-category profile is descriptive.

Supplementary Table S8A. Exploratory 'Profilaktyka 40+' programme responses

Programme characteristic	n/N	%
**No advice**	46/70	65.7
**Brief guidance**	20/70	28.6
**Detailed information**	4/70	5.7
**Diet change**	16/70	22.9
**Started supplementation**	19/70	27.1
**Increased activity**	18/70	25.7
**No lifestyle change**	31/70	44.3
**Changed supplementation**	27/70	38.6
**Programme influenced supplementation approach**	31/70	44.3

Programme analyses are exploratory and cross-sectional.

Supplementary Table S8B. Exploratory associations with receiving 'Profilaktyka 40+' nutrition/supplement advice

Outcome	Fisher OR	raw p	BH q
**Any lifestyle change**	7.11	0.0009	0.0028
**Supplementation change**	2.70	0.0715	0.0715
**Influenced supplementation approach**	4.13	0.0107	0.0160

Associations do not establish programme effects or temporal causality.

Supplementary Table S8C. Exploratory association between 'Profilaktyka 40+' programme participation and current supplement use

Comparison	Current supplement use	Reference group	aOR	95% CI	p
Programme participant vs. nonparticipant	60/70 (85.7%)	53/80 (66.3%)	3.37	1.44–7.88	0.005

Exploratory adjusted logistic regression controlling for age, sex, higher education, chronic disease, and regular medication use. These cross-sectional results are associative and do not establish an effect of programme participation on supplement use.

Supplementary Table S9. Latent-class model selection and revision-stage indicator-specification robustness

Specification	Classes	BIC	AIC	Relative entropy	Assigned class sizes
**Original 7 indicators**	1	930.69	911.92	—	108

**Original 7 indicators**	2	892.40	852.17	0.784	61, 47
**Original 7 indicators**	3	913.80	852.11	0.750	13, 40, 55
**Original 7 indicators**	4	942.64	859.50	0.800	37, 15, 51, 5
**Remove P28 composite**	1	793.06	776.97	—	108
**Remove P28 composite**	2	759.69	724.82	0.800	70, 38
**Remove P28 composite**	3	778.04	724.40	0.889	15, 67, 26
**Remove P28 composite**	4	806.05	733.63	0.796	30, 23, 15, 40
**Remove P29 professional source**	1	776.44	760.35	—	108
**Remove P29 professional source**	2	775.02	740.15	0.681	65, 43
**Remove P29 professional source**	3	795.34	741.69	0.769	47, 54, 7
**Remove P29 professional source**	4	818.73	746.31	0.829	52, 4, 5, 47
**Remove both overlap-linked indicators**	1	638.81	625.40	—	108
**Remove both overlap-linked indicators**	2	644.93	615.43	0.593	45, 63
**Remove both overlap-linked indicators**	3	662.51	616.92	0.824	15, 31, 62
**Remove both overlap-linked indicators**	4	686.47	624.78	0.853	62, 15, 2, 29

All robustness specifications were fitted in the same 108 participants complete for the original seven-indicator set. The original two-class model had the lowest BIC under the original specification, but evidence for a two-class structure weakened substantially after removal of the overlap-linked P28/P29 indicators; when both were removed, BIC favored one class. LCA is therefore retained only as exploratory/hypothesis-generating.

Supplementary Figure S1. Item-level factual supplement-safety knowledge.

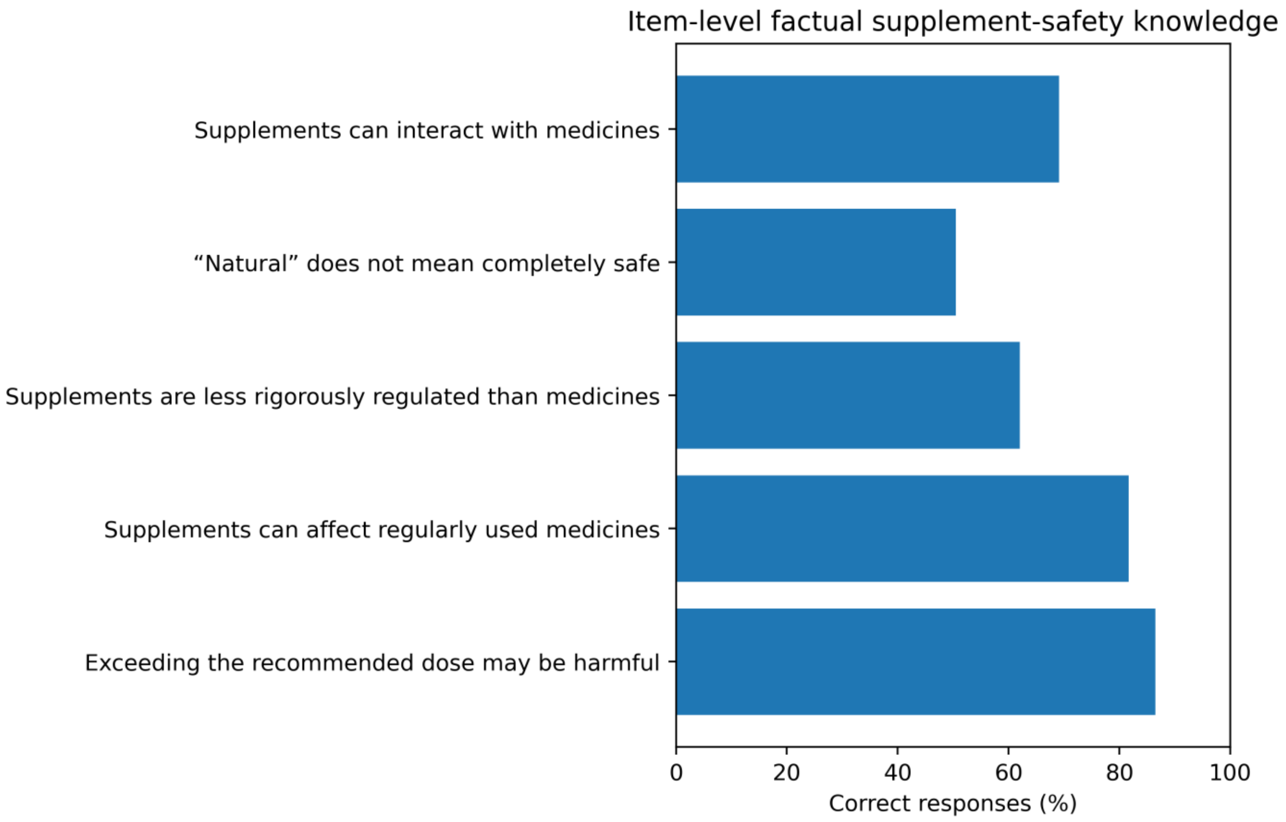

Percent correct uses item-specific denominators.

Supplementary Figure S2. Reported dietary supplement categories among current users.

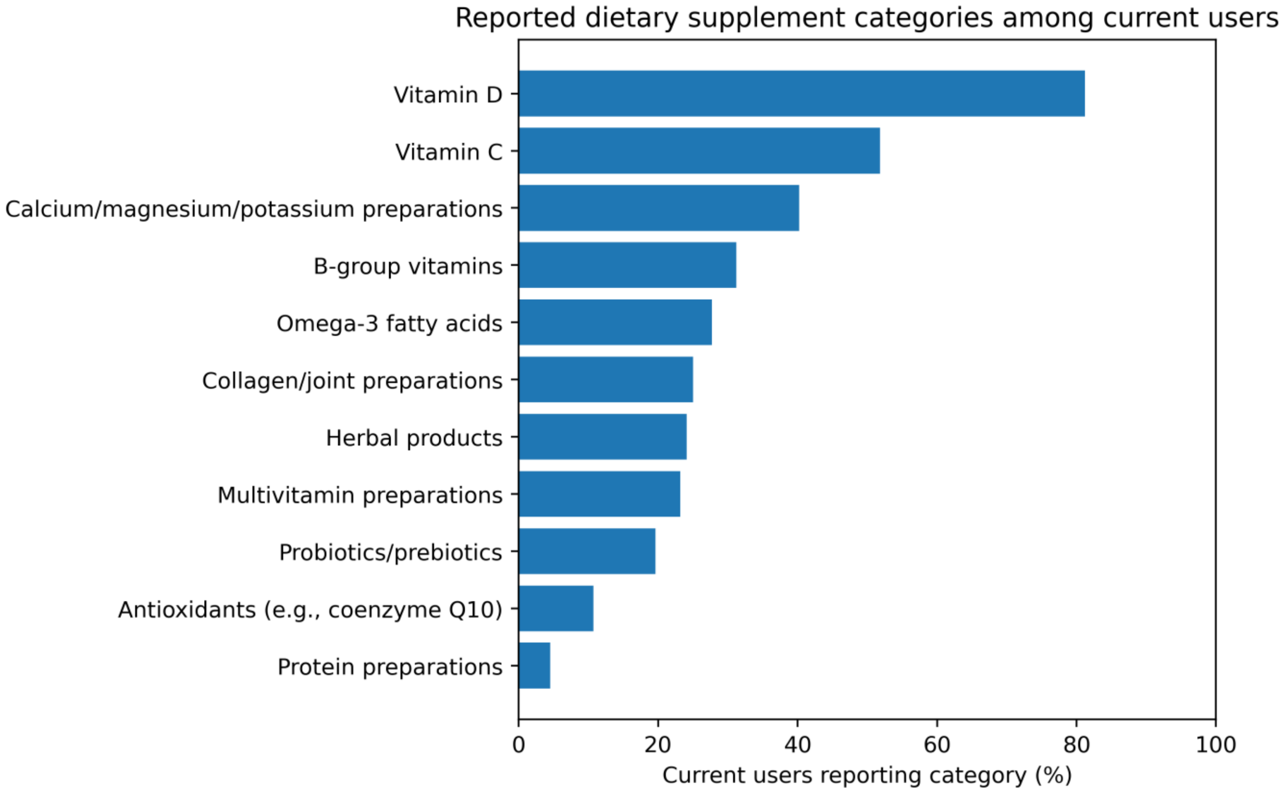

Broad categories are descriptive and are not a clinical risk classification.

Supplementary Figure S3. Exploratory associations with receiving 'Profilaktyka 40+' nutrition/supplement advice.

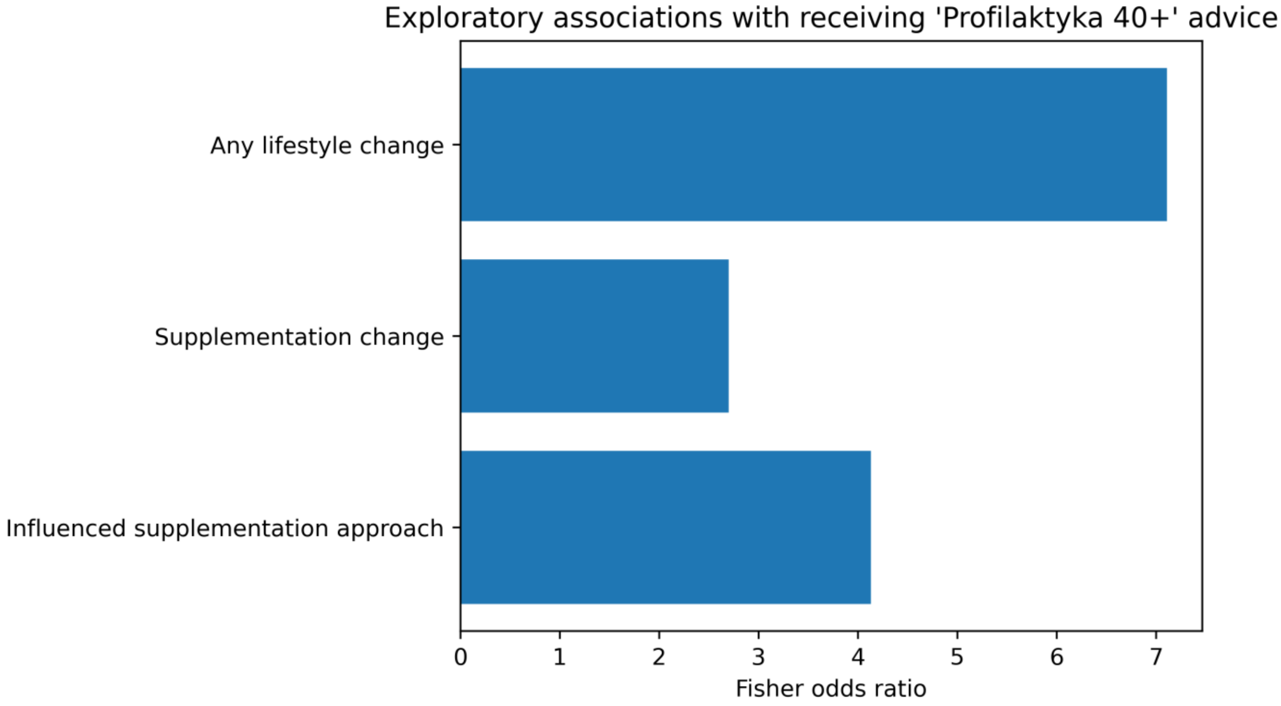

Fisher odds ratios; see Supplementary Table S8B for raw p and BH q values.

Supplementary Figure S4. Original exploratory two-class behavioural solution.

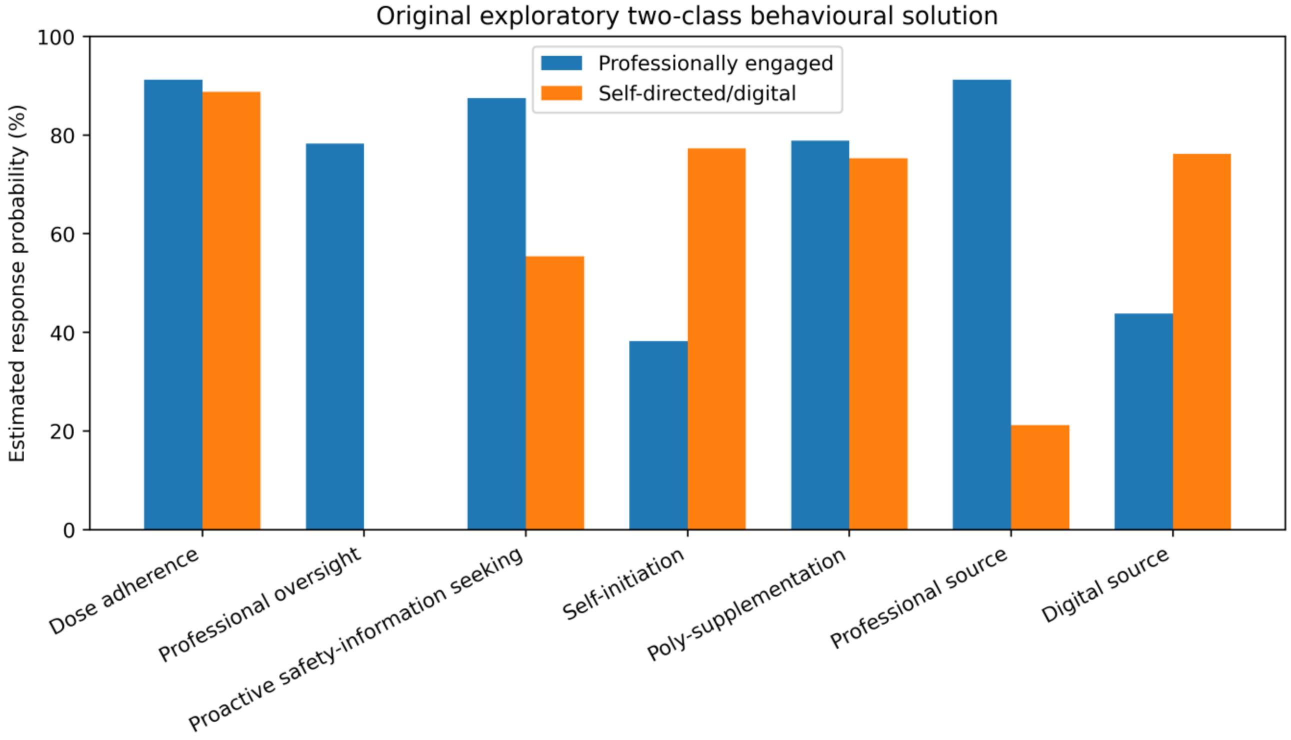

Shown for transparency. Revision-stage robustness analyses demonstrated sensitivity to indicator specification; see Supplementary Table S9.

Supplementary File S1. Complete questionnaire - English reporting translation

The questionnaire contained 46 numbered questions and was administered in Polish. The English text below is a reporting translation reconstructed from the source instrument fields and response options in the frozen dataset. It is provided to permit readers to inspect the complete measurement instrument and coding context. This English reporting translation has not undergone independent forward-backward translation or validation and should not be treated as a validated English-language questionnaire.

1. Informed consent

Do you consent to participate in the study?

- Yes
- No

2. Age eligibility

Are you 40 years of age or older?

- Yes
- No

3. Sex

Sex.

- Female
- Male

4. Age

Age (years).

- Open numeric response

5. Height

Height (cm).

- Open numeric response

6. Body weight

Body weight (kg).

- Open numeric response

7. Place of residence

Place of residence.

- Village

- Town/city up to 20,000 inhabitants
- Town/city 20,000–100,000 inhabitants
- City >100,000 inhabitants

8. Education

Education.

- Primary
- Vocational
- Secondary
- Higher education (bachelor/engineer/master)

9. Marital status

Marital status.

- Single
- Married
- Widowed
- In a relationship

10. Current life/work situation

Current life/work situation.

- On-site work
- Remote work
- Physical work
- Retired/disability pension
- Unemployed
- Other/free text

11. Material situation

How do you assess your financial/material situation?

- Very good
- Good
- Average
- Rather poor

12. Chronic disease

Do you have a chronic disease (e.g., diabetes, hypertension, thyroid disease, other)?

- Yes
- No
- If yes: open-text description

13. Regular medication

Do you take any medications regularly?

- Yes
- No
- If yes: open-text medication description

14. Physical-activity profile

Please indicate the description that best reflects your physical activity.

- Sedentary lifestyle/office work, no exercise
- Sedentary lifestyle/office work, physical activity 2–3 times/week
- Moderately active lifestyle, no structured activity
- Moderately active lifestyle, daily physical activity
- Physical work/high physical effort

15. ≥30-min physical activity

How often do you undertake physical activity lasting at least 30 minutes?

- Every day
- 4–6 times/week
- 2–3 times/week
- Once/week
- Less than once/week
- Never

16. Main physical activities

Which types of physical activity do you undertake most often? (select all that apply)

- Walking/marching
- Cycling (stationary or traditional)
- Nordic walking
- Running
- Swimming
- Strength training
- Fitness/group classes
- Home exercise
- Yoga
- None
- Other/free text

17. Physical-activity attitudes

To what extent do you agree with each statement about physical activity?

- Statements: Regular physical activity is crucial for health after age 40; I try to remain physically active despite responsibilities; Physical activity improves my well-being; I believe physical activity can reduce the need for supplementation; I would like to increase my physical activity
- Response scale: strongly disagree / disagree / neither agree nor disagree / agree / strongly agree

18. Current supplement use

Do you currently use dietary supplements? If "Never", proceed to Question 33.

- Yes – regularly (at least once per week)
- Yes – occasionally (less than once per week)
- No, but I used them in the past
- Never used

19. Number used simultaneously

How many dietary supplements do you use simultaneously?

- 1
- 2–3
- 4–5
- More than 5

20. Supplement categories

Which dietary supplements do you currently use or have you used within the last 12 months? (select all that apply)

- Vitamin D
- Vitamin C
- B-group vitamins
- Multivitamin/multivitamin complex
- Omega-3 fatty acids (fish oil/EPA/DHA)
- Calcium/magnesium/potassium preparations
- Probiotics/prebiotics
- Protein preparations
- Collagen/joint preparations
- Herbal products (examples in original item included ginseng, ginkgo, ashwagandha/berberine)
- Antioxidants (e.g., coenzyme Q10)
- Other/free text

21. Supplement frequency

How often do you take the indicated dietary supplements?

- Daily
- Several times per week
- Seasonally only

- Occasionally
- I do not remember/varies

22. Duration of use

How long have you used dietary supplements (regularly or occasionally)?

- <6 months
- 6–12 months
- 1–3 years
- >3 years

23. Initiator

Who initiated your use of dietary supplements? (select all that apply)

- Physician after medical consultation
- Pharmacist
- Dietitian/nutrition specialist
- Trainer/personal trainer
- Myself/own decision
- Family/friends
- Advertising/social media/influencer
- Other/free text

24. Reasons for use

What are the main reasons for using dietary supplements? (select all that apply)

- To supplement vitamins/minerals in the diet
- Improve immunity
- Improve well-being/increase energy
- Prevention of age-related diseases
- Improve memory/concentration
- Laboratory results/deficiencies
- Physician/dietitian recommendation
- Improve appearance (skin/hair/nails)
- Other/free text

25. Dose adherence

Do you follow the recommended dose?

- Always
- Often
- Sometimes
- Never

26. Oversight

Who monitors the appropriateness of your supplementation? (select all that apply)

- Treating physician/specialist physician
- Primary-care physician/family doctor or pharmacist
- Clinical dietitian/nutrition specialist
- Other medical professional
- Nobody – I manage supplementation myself
- Family/friends
- Internet/social media
- Other/free text

27. Discontinuation after adverse effects

Have you ever stopped taking a dietary supplement because of adverse effects?

- Yes
- No
- If yes: open-text description

28. Safety-information seeking

Have you sought information about possible interactions between the dietary supplements you use and medicines, and about their adverse effects? (select all that apply)

- Yes – I consulted a physician
- Yes – I asked at a pharmacy
- Yes – I searched the internet/leaflet
- No – I did not seek information

29. Supplement information sources

Where do you most often obtain information about dietary supplements? (select all that apply)

- Physician
- Pharmacist
- Dietitian/nutrition specialist
- Internet articles/social media
- Online forums/groups
- Advertising/radio/press/pharmacy leaflets
- Family/friends
- Other/free text

30. Trust in information sources

Rate your trust in each source.

- Sources: physician; pharmacist; dietitian; websites/forums; influencers/social media; advertising
- Response scale: definitely do not trust / rather do not trust / neutral / rather trust / definitely trust

31. Factual supplement-safety statements

Please answer True, False, or Don’t know for each statement.

- 1. Dietary supplements do not interact with medicines, so they can be safely taken regardless of medicines used.
- 2. A “natural” ingredient means that a dietary supplement is completely safe.
- 3. Dietary supplements are not regulated as rigorously as medicines.
- 4. A supplement label should contain information on the recommended dose and possible contraindications for use. [Not included in publication score because compound wording did not permit unambiguous binary correctness scoring.]
- 5. Dietary supplements can affect the action of medicines taken regularly.
- 6. Exceeding the recommended dose of a dietary supplement may be harmful.

32. Supplement-related attitudes and self-perceptions

To what extent do you agree with each statement?

- Dietary supplements are necessary to maintain health after age 40.
- A physician should always be consulted before starting supplementation.
- I always check the composition and recommended dose before purchase.
- My diet alone is sufficient to meet vitamin/mineral requirements.
- Dietary supplements can be dangerous if used without consultation with a specialist.
- I am concerned about using supplements without prior laboratory tests.
- Supplements should be monitored in the same way as medicines.
- Most supplements available on the market are effective.
- Supplement advertisements are a credible source of information for me.
- I believe supplements can significantly improve my well-being.
- I choose supplements because they are easier to use than changing my diet.
- I use supplements when I feel tired or low in energy.
- Regular supplementation is an important part of caring for health after age 40.
- I can assess whether a supplement is of good quality.
- I feel well informed about the effects of the supplements I use.
- Many supplements have unconfirmed effects.
- Participation in preventive programmes (e.g., Profilaktyka 40+) helps people make informed supplementation decisions.
- Consultation with a dietitian is crucial before starting supplementation.
- I often follow the opinions of others (family/friends) regarding supplements.
- Social media influence my supplementation decisions.
- Response scale: strongly disagree / disagree / neither agree nor disagree / agree / strongly agree

33. Nutrition knowledge self-rating

How do you assess your knowledge of healthy eating?

- Very good
- Good
- Average
- Poor

34. Diet balance

Do you think your diet is sufficiently balanced?

- Yes
- No
- Difficult to say

35. Interest in healthy eating

Are you interested in healthy eating?

- Yes, I regularly follow information
- Sometimes
- Rarely
- Not at all

36. Following a balanced diet

Do you follow the principles of a balanced diet?

- Yes, consistently
- I try, but not always
- Rarely
- No

37. Diet self-rating

How do you assess your diet?

- Healthy and balanced
- Relatively healthy but with errors
- Difficult to say
- Not very healthy

38. Food composition attention

How often do you pay attention to the composition of food products?

- Always
- Often
- Rarely

- Never

39. Dietitian consultation

Have you ever used a dietitian consultation?

- Never
- Once
- Several times
- Yes, regularly

40. Sources of healthy-eating knowledge

Where do you obtain information on how to balance your diet properly? (select all that apply)

- Internet health portals/blogs/forums
- Social media (Instagram, TikTok, etc.)
- Dietitian/nutrition specialist
- Physician
- Family/friends
- Books and scientific publications
- Press articles/health magazines
- Courses/training/webinars on healthy eating
- Advertising/promotional materials
- I am not interested/do not seek knowledge
- Other/free text

41. Profilaktyka 40+ participation

Have you participated in the "Profilaktyka 40+" programme? If "No", proceed to the end of the questionnaire.

- Yes
- No

42. Reasons for programme participation

What motivated you to participate in "Profilaktyka 40+"? (select all that apply)

- Desire to monitor health status
- Free tests
- Physician recommendation
- Health problems
- Curiosity about results
- Other/free text

43. Programme advice

During participation in "Profilaktyka 40+", did you receive advice on nutrition and supplementation?

- No
- Yes – brief guidance
- Yes - detailed information

44. Lifestyle changes after programme

After participating in “Profilaktyka 40+”, did you make lifestyle changes? (select all that apply)

- Yes - changed diet
- Yes - started supplementation
- Yes - increased physical activity
- No - changed nothing
- Other/free text

45. Supplementation change after programme

Did you change your dietary supplementation after participating in “Profilaktyka 40+”?

- Yes
- No

46. Programme influence on supplementation

Did the test results from “Profilaktyka 40+” influence your approach to dietary supplementation?

- No - no influence
- Yes - I reduced supplementation
- Yes - I pay more attention to dietary deficiencies
